# Preparation and Characterization of AMT/Co(acac)_3_-Loaded PAN/PS Micro-Nanofibers with Large through-Pores

**DOI:** 10.1186/s11671-019-3059-y

**Published:** 2019-08-20

**Authors:** Fei-Fei Wang, Hui-Mei Zhang, Qian Wang, Cui-Cui Fang, Rong Zhang, Ping Wang, Yan Zhang

**Affiliations:** National Engineering Laboratory for Modern Silk, College of Textile and Clothing Engineering, Soochow University, Suzhou, China

**Keywords:** Electrospinning, Porous, Large thorough-pore, Micro-nanofibers, Preparation

## Abstract

This study focused on the fabrication and characterization of ammonium metatungstate hydrate (AMT) combined with cobalt(III) acetylacetonate (Co(acac)_3_)-loaded electrospun micro-nanofibers. The morphologies, structures, element distribution, through-pore size, and through-pore size distribution of AMT/Co(acac)_3_-loaded PAN/PS micro-nanofibers were investigated by a combination of field emission scanning electron microscopy (FESEM), flourier transformation infrared (FTIR) spectroscopy, energy disperse spectroscopy (EDS), through-pore size analyzer, and so on. These micro-nanofibers have many advantages in their potential application as electro-catalysts. The porous and large thorough-pore will benefit for effective electrolyte penetration, in addition to promoting gas bubbles evolving and releasing from catalyst surface timely.

## Introduction

Nanofiber is a unique class of nanomaterials with many interesting properties owing to their nanoscale diameters and large aspect ratio. They possess excellent mechanical properties, and their surface can be readily modified due to their high surface area to volume ratio [1]. Electrospinning was rapidly emerging as a simple and reliable technique for the preparation of smooth nanofibers with controllable morphology from a variety of polymers [2–5]. Meanwhile, theories like thermo-electro-hydrodynamic model [6] were built to support this technology. Li [7] and Liu [8] fabricated fibers containing Cu and Fe_2_O_3_ via bubble electrospinning. Lin et al. [9] fabricated core-shell nanofibers with PS as core and PAN as shell, and Wu et al. [10] explored the effect of three polymer systems, Poly(m-phenylene isophthalamide) (Nomex)/polyurethane (TPU), polystyrene (PS)/TPU, and polyacrylonitril (PAN)/TPU, on the formation process of helical nanofibers via co-electrospinning.

The ammonium metatungstate hydrate (AMT) and cobalt(III) acetylacetonate (Co(acac)_3_) can be usually used as additive in solution. Arman et al. [11] added polyoxometallate and AMT to the electro-winning of copper from synthetic copper sulphate solutions and found that AMT can be used as an additive for reducing power consumption in electro-winning of copper from sulphate solutions. Petrov et al. [12] presented a result of laboratory experiments on modeling of heavy oil oxidation processes in an air-oxygen environment using Co(acac)_3_ as a catalyst at certain temperature and pressure which was typical thermal production method. Xu et al. [13] carried out reversible addition-fragmentation chain-transfer polymerization of acrylonitrile using Co(acac)_3_ as an initiator which was achieved at 90 °C and mediated by 2-cyanoprop-2-yl dithionaphthalenoate. In another research [14], iron- and cobalt-incorporated carbon nanofibers (FeCo-CNFs) were prepared as a substitute of Pt-based electrocatalyst via electrospinning of PAN solution containing iron(III) acetylacetonate and cobalt(II) acetylacetonate and subsequent pyrolysis of the blend precursor fibers.

In this article, we demonstrated an efficient process to fabricate the AMT/Co(acac)_3_-loaded PAN/PS micro-nanofibers with porous and large through-pore structure through one-step electrospinning technique. First, the PAN/PS fibers were carefully designed by tuning the concentration of PAN/PS in DMF. Then, by regulating the molar ratios (W^6+^:Co^3+^) in the PAN/PS solution, the AMT/Co(acac)_3_-loaded PAN/PS micro-nanofibers were fabricated. Moreover, the morphologies, structures, element distribution, through-pore size, and through-pore size distribution of these fibers were systematically investigated. Due to the porous and large through-pore favor effective electrolyte penetration, in addition to promoting gas bubbles evolving and releasing from catalyst surface timely, these micro-nanofibers might have potential applications in electrochemical reaction.

## Methods

### Materials

Polyacrylonitrile (PAN, (C_3_H_3_N)_*n*_, *M*_*w*_ = 150,000 g mol^−1^, Shanghai Macklin Biochemical, Co., Ltd., China), polystyrene (PS, [CH_2_CH(C_6_H_5_)]_*n*_, *M*_*w*_ = 192,000 g mol^−1^, Sigma-Aldrich), *N*,*N*-dimethylformamide (DMF, HCON(CH_3_)_2_, *M*_*w*_ = 73.09 g mol^−1^, AR, Chinasun Specialty Products, Co., Ltd., China), ammonium metatungstate hydrate (AMT, (NH_4_)_6_H_2_W_12_O_40_·XH_2_O, *M*_*w*_ = 2956.30 g mol^−1^, 99.5% purity, Shanghai Macklin Biochemical, Co., Ltd., China), and cobalt(III) acetylacetonate (Co(acac)_3_, C_15_H_21_CoO_6_, *M*_*w*_ = 356.26 g mol^−1^, 99.5% purity, Sigma-Aldrich). All materials were used as received without any further purification.

### Fabrication of AMT/Co(acac)_3_-Loaded PAN/PS Micro-Nanofibers

**Scheme 1:**
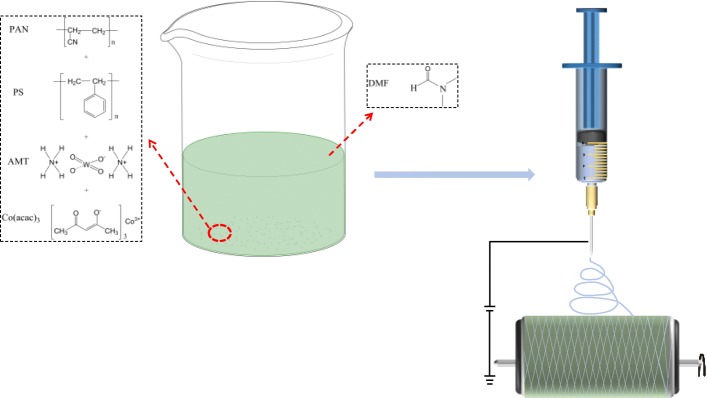
Schematic illustration of the preparation process for the micro-nanofibers

All concentration measurements were done in weight by weight (*w*/*w*). PAN/PS solutions at concentrations ranging from 10 to 20 wt% were prepared by dissolving a mixture of PAN/PS (1/1, *w*/*w*) in DMF with stirring to obtain a homogeneous solution at room temperature. The AMT and Co(acac)_3_ were then added into the above 20 wt% mixed solution (1.25 g PAN, 1.25 g PS, 10 ml DMF, PAN/PS-20 wt%) with five different amounts of tungsten and cobalt ions, which are 1:0 (W^6+^ = 0.62 mmol), 0:1 (Co^3+^ = 0.62 mmol), 1:1 (W^6+^ = 0.62 mmol, Co^3+^ = 0.62 mmol), 1:2 (W^6+^ = 0.62 mmol, Co^3+^ = 1.24 mmol), and 1:3 (W^6+^ = 0.62 mmol, Co^3+^ = 1.87 mmol) stirred overnight (denoted as PAN/PS W_1_, PAN/PS Co_1_, PAN/PS W_1_Co_1_, PAN/PS W_1_Co_2_, and PAN/PS W_1_Co_3_). The PAN/PS shown in this paper defaults to a concentration of 20 wt%. The resulting uniform solution was filled into a 10 mL syringe, and a grounded metallic rotating roller (Changsha Nai Instrument Technology, Co., Ltd., China) was used as the collector. A high voltage of 20 kV was applied between the needle tip and collector, and the spinning rate was controlled at 1 ml/h. All experiments were carried out at room temperature (20 ± 3 *°C*) and a relative humidity of 40 ± 3%. The schematic illustration of the preparation process for the micro-nanofibers was shown in Scheme 1.

### Measurements and Characterizations

#### Fiber Morphology

The morphologies and structures of as-made electrospun fibers were examined by a field emission scanning electron microscopy (FESEM, Hitachi S-4800, Japan). All samples were dried at room temperature and then sputter-coated with gold by an IB-3 (Eiko, Tokyo, Japan) for 90 s. ImageJ software (National Institute of Mental Health, Bethesda, Maryland, USA) was applied for fiber diameter characterization with 100 fibers chosen randomly from the FESEM pictures.

#### Fiber Chemical Structure

The chemical structure of fibers was investigated through FTIR spectroscopy (Nicolet5700, Thermo Nicolet Company, Madison, Wisconsin, USA) at room temperature by KBr squashed. The spectrum was got by the performance of 32 scans with the wavenumber ranging from 400 to 4000 cm^−1^.

#### Fiber Elemental Detection

Energy disperse spectroscopy (EDS) was conducted by using a TM3030 scanning electron microscopy to detect the element and the relative surface distribution of AMT/Co(acac)_3_ on the fiber membrane and the randomly selected areas.

#### Through-Pore Properties

The through-pore size and through-pore size distribution of fibrous membrane were measured using through-pore size analyzer (Porometer 3G, Quantachrome Instruments, USA). All samples were cut to circular membranes with a diameter of 25 mm and thoroughly wetted with a Liquid Accessory Kit (Number 01150-10035) to fill all pores with liquid.

## Results and Discussion

### Morphological Characterization of PAN/PS Fibers

**Fig. 1:**
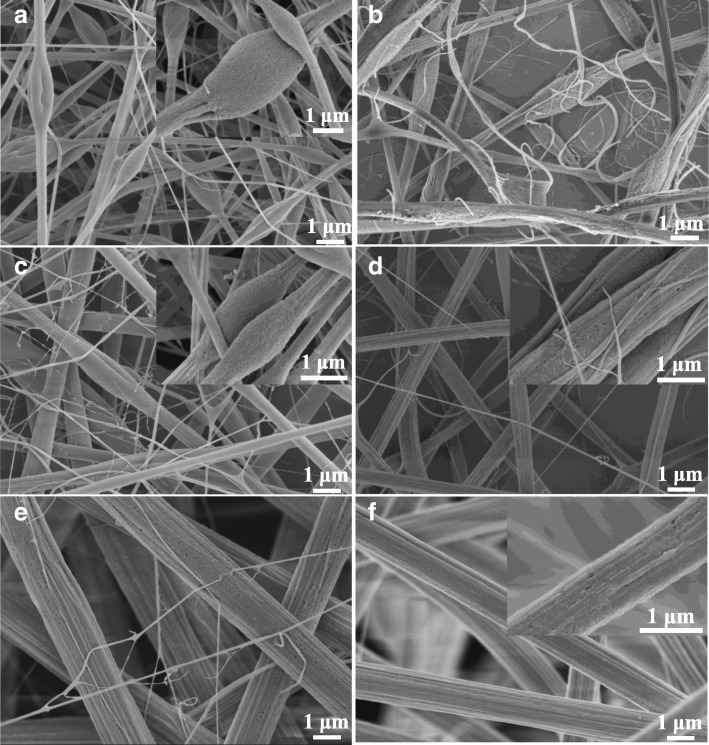
FESEM pictures PAN/PS fibers with different concentration. **a** PAN/PS-10 wt%, **b** PAN/PS-12 wt%, **c** PAN/PS-14 wt%, **d** PAN/PS-16 wt%, **e** PAN/PS-18 wt%, **f** PAN/PS-20 wt%

**Fig. 2:**
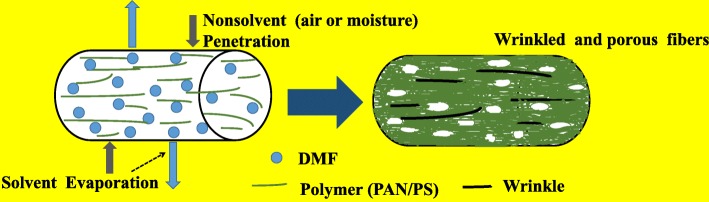
Schematic diagrams illustrating the formation process of wrinkled and porous fiber in electrospinning

**Fig. 3:**
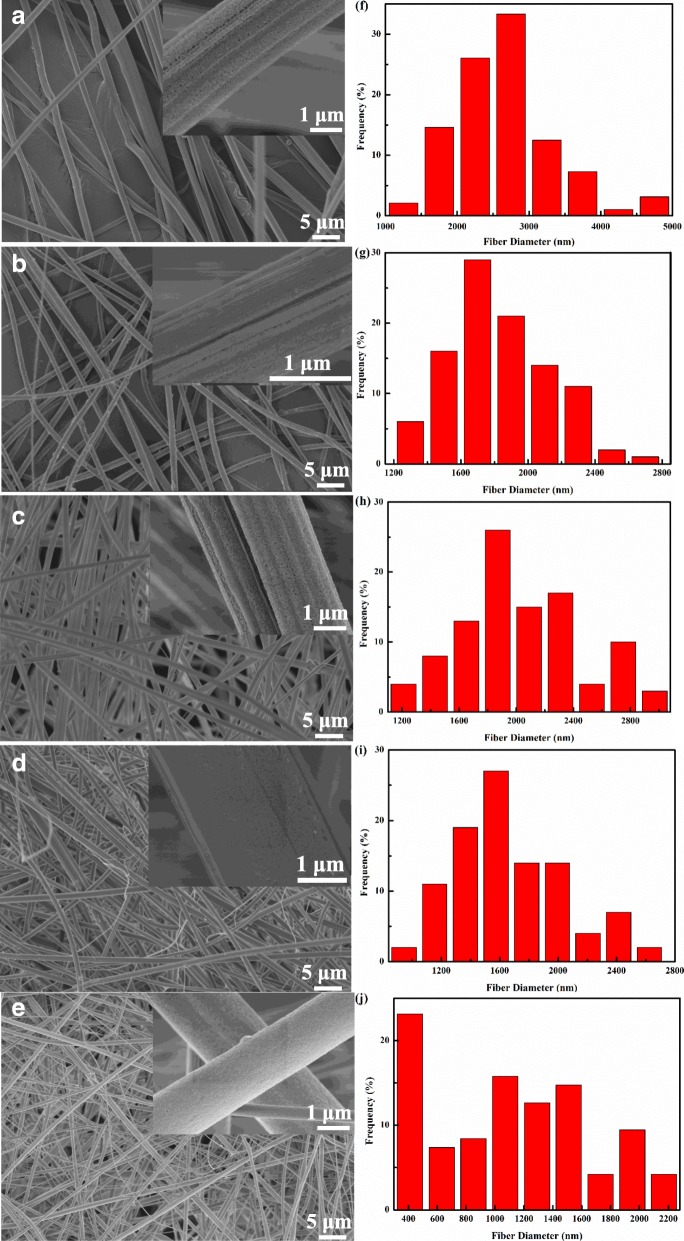
FESEM pictures and fiber diameter distribution of micro-nanofibers with different molar ratio (W^6+^:Co^3+^). **a**, **f** PAN/PS W_1_, **b**, **g** PAN/PS Co_1_, **c**, **h** PAN/PS W_1_Co_1_, **d**, **i** PAN/PS W_1_Co_2_, **e**, **j** PAN/PS W_1_Co_3_

Concentration is reckoned as one of the most important parameters keeping fiber morphology in functional order [15–17]. FESEM images of the PAN/PS fibers prepared from various concentrations of PAN/PS solutions in DMF are shown in Fig. 1. Figure 1a shows a typical FESEM image of the PAN/PS-10 wt%, from which it can be seen that a large number of scattered filaments and micron and submicron-sized beads along the fiber axis, and wrinkled and porous surface on the beads or fibers. Vapor phase separation may be the main reason for such a porous and wrinkled morphology [18, 19], as shown in Fig. 2. When the mixture solution of PAN and PS sprayed from injector, the ambient temperature declined sharply due to the quick volatilization of DMF which induced phase separation of solution. After that, the jet flow can be divided into rich solute phase which solidified to matrix and rich solvent phase which gasified to pores. Moreover, the jet flow was also drawn in the air under the combined force of electric field and surface tension of solution. Due to the difference of solidification mode between PAN and PS, the wrinkle formed in the surface of fibers resulting from mutual squeeze between polymers and air. When the concentration of the PAN/PS solution was increased to 12 wt% and 14 wt%, the beads on the fibers become longer and thinner-like spindle, and the scattered filament still exists, as shown in Fig. 1b, c. At PAN/PS-16 wt% (Fig. 1d), the micron and submicron-sized beads almost disappear, and scattered filaments began to coexist with the ordered filaments. PS is a necessary ingredient to these ordered filaments. When the concentration increased to 18 wt% and above, structures of pure fiber were obtained (Fig. 1e, f). Moreover, structures of scattered filament were almost disappeared, which corresponds to the concentration of 20 wt% (Fig. 3(f)). These changes from beads to pure fibers could be attributed to the solution viscosity caused by the concentration. The formation of beads was attributed to an insufficient stretch of the filaments during the whipping of the jet [20]. However, a higher viscoelastic force helped suppress the surface tension of the polymer solution and contributed to the generation of bead-less fibers [21].

### Morphological Characterization of AMT/Co(acac)_3_-Loaded PAN/PS Micro-Nanofibers

Figure 3 shows the FESEM images as well as fiber diameter distribution of AMT/Co(acac)_3_-loaded PAN/PS micro-nanofibers obtained by varying the molar ratios (W^6+^:Co^3+^) while the other parameters were kept constant. It was observed that all of the fibers showed some nanopores on their surfaces, which was due to the phase separation mentioned in the previous section [18, 19]. By increasing the molar ratios (W^6+^:Co^3+^) in the PAN/PS solution, the surface structure of the AMT/Co(acac)_3_-loaded PAN/PS micro-nanofibers was remarkably changed from ordered filaments to scattered filaments and the fiber diameters of PAN/PS W_1_, PAN/PS Co_1_, PAN/PS W_1_Co_1_, PAN/PS W_1_Co_2_, and PAN/PS W_1_Co_3_ changed from 2765.21 ± 180.44, 1832.83 ± 56.73, 2031.57 ± 82.65, and 1671.35 ± 75.67 to 1092.02 ± 111.71 nm, which could be ascribed to the addition of AMT and Co(acac)_3_.

### FTIR Analysis

**Fig. 4:**
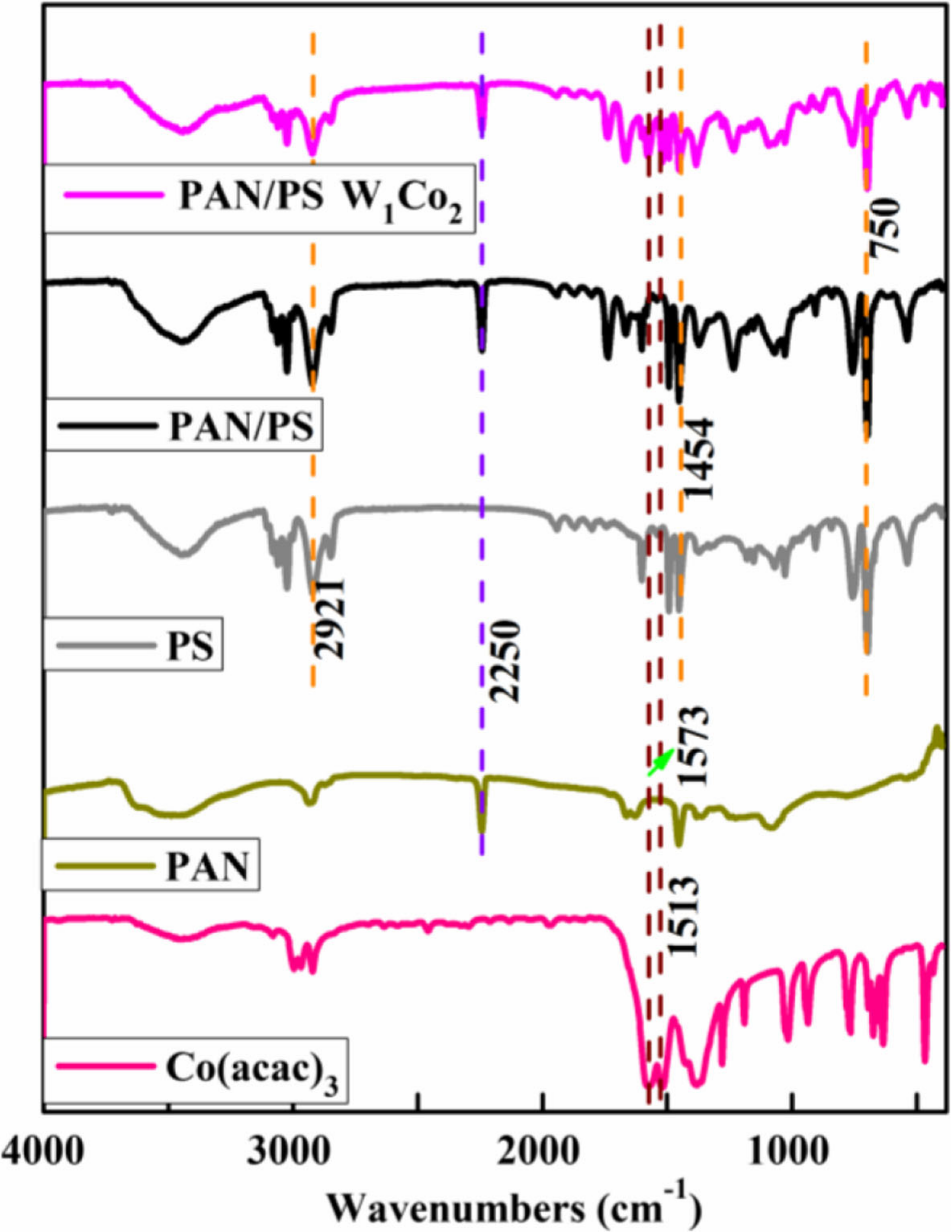
Fourier-transform infrared (FTIR) spectra

Figure 4 shows the FTIR spectra for pure Co(acac)_3_, PAN fibers, PS fibers, PAN/PS fibers, and corresponding micro-nanofibers containing AMT and Co(acac)_3_. The spectrum of the Co(acac)_3_ exhibited asymmetric and symmetric stretching vibrations of the chelate group (C=C) and (C=O + C–H) which appeared at 1573 and 1513 cm^−1^, respectively [22–24]. The intensity of the nitrile peak of PAN showed peaks at 2250 cm^−1^, which was due to the presence of C≡N [9]. Those band at 750, 2921, and 1454 cm^−1^ were ascribed to the C–H out plane normal vibrations of the phenyl groups, CH_3_ asymmetric stretching and bending vibrations by PS [23]. The results showed Co(acac)_3_, PAN and PS were successfully fabricated to the micro-nanofibers. This would be further proved by EDS in the succeeding section.

### EDS Test

**Fig. 5:**
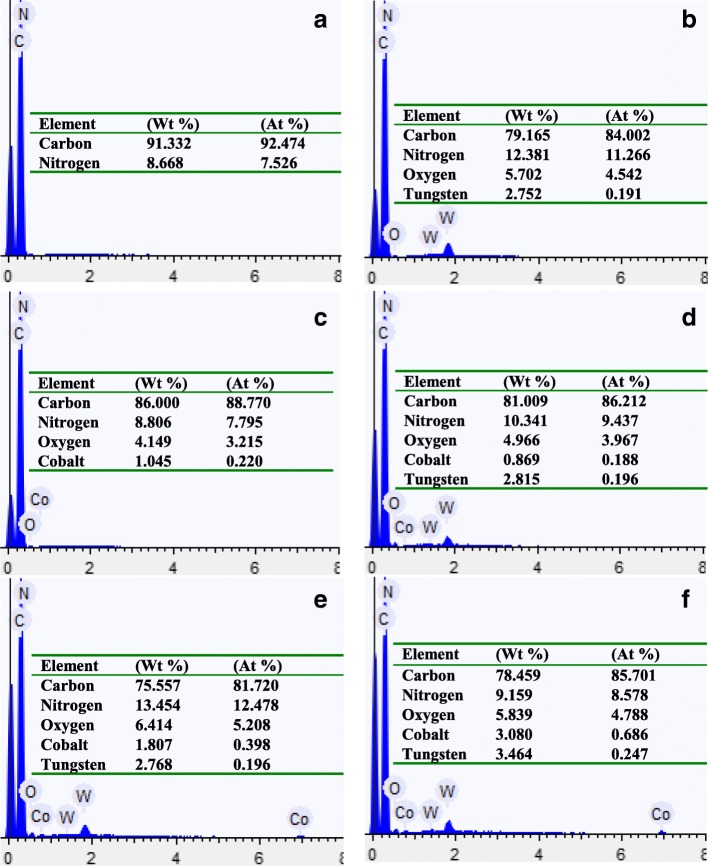
EDS of **a** PAN/PS, **b** PAN/PS W_1_, **c** PAN/PS Co_1_, **d** PAN/PS W_1_Co_1_, **e** PAN/PS W_1_Co_2_, **f** PAN/PS W_1_Co_3_

**Fig. 6:**
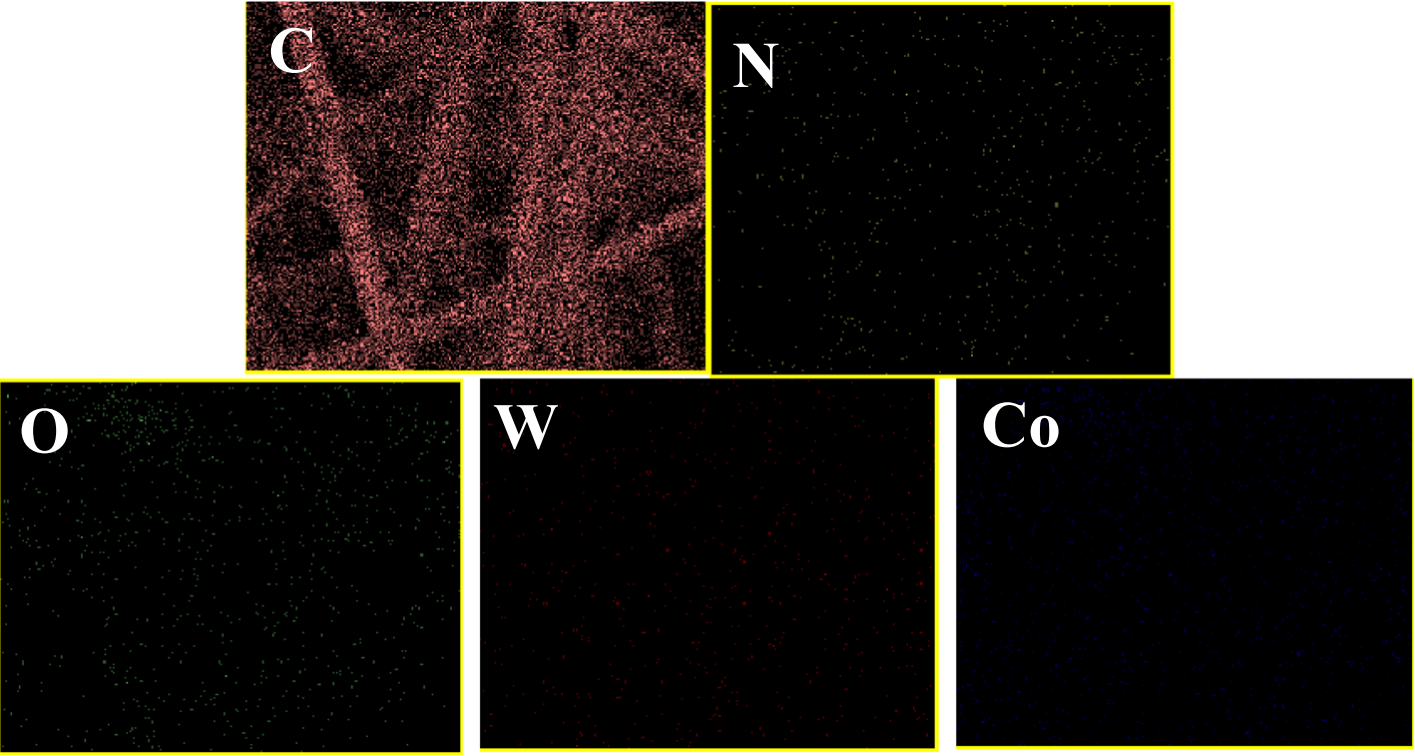
Element mapping images of PAN/PS W_1_Co_3_

Energy disperse spectroscopy (EDS) analysis was used to investigate the chemical composition and relative abundance of AMT/Co(acac)_3_ on the surface of PAN/PS fiber membranes. The elemental composition of the relevant membrane was determined by the EDS spectrum of the randomly selected area (Figs. 5 and 6). The table insets in the figures list the atomic ratio and weight ratio of the detected elements of the relevant fiber membranes. The main related elements are carbon (C), nitrogen (N), oxygen (O), cobalt (Co), and tungsten (W). No W, Co, and O peaks appeared in the spectrum of the pure PAN/PS fiber membrane, no Co peak appeared in the spectrum of the PAN/PS W_1_, and no W peak appeared in the spectrum of the PAN/PS Co_1_, while the Co content in spectrum for AMT/Co(acac)_3_-loaded PAN/PS micro-nanofibers increased as the molar ratios (W^6+^:Co^3+^) increased and proved the molar ratio in the initial experiment design. The EDS result further confirmed that the AMT and Co(acac)_3_ were both successfully loaded onto the PAN/PS fibers.

### Through-Pore Structures

**Fig. 7:**
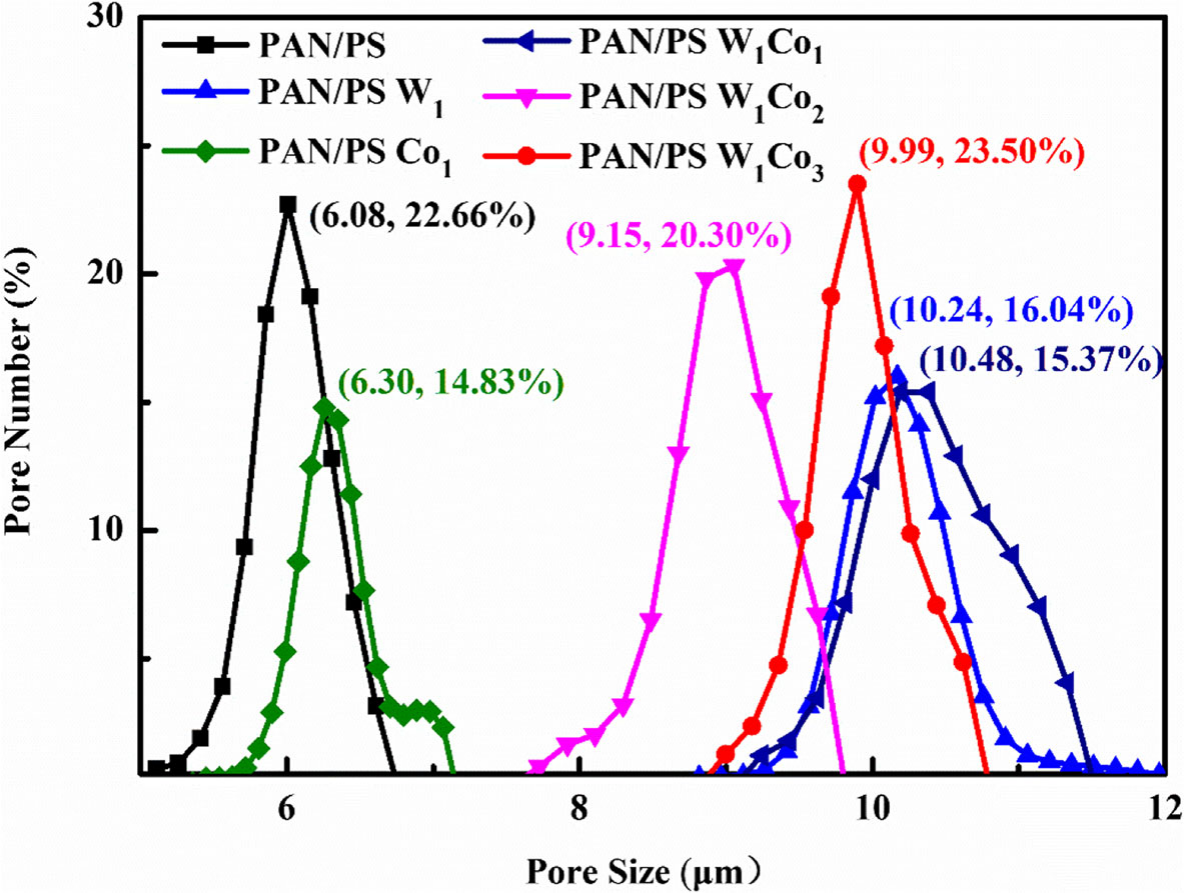
Through-pore size distributions of micro-nanofibers membranes

**Fig. 8:**
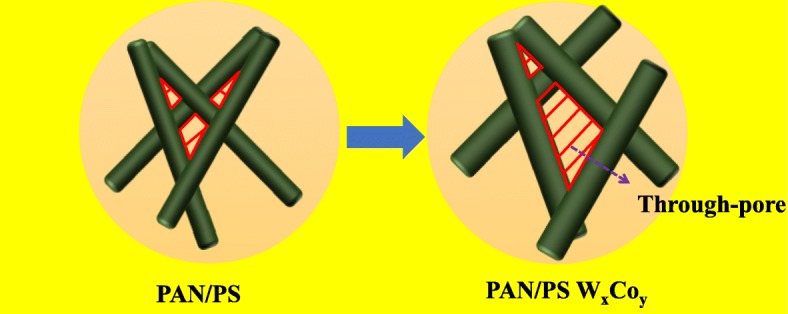
Schematic of through-pore change of AMT/Co(acac)_3_-loaded PAN/PS micro-nanofibers

The average thorough-pore size and through-pore size distribution, which are very important parameters to determine the electrolyte penetration, gas bubbles evolving, and releasing from catalyst surface timely, of the AMT/Co(acac)_3_-loaded PAN/PS micro-nanofibers at the relative molar ratios (W^6+^:Co^3+^) were measured through a through-pore size analyzer as shown in Fig. 7, and the average through-pore sizes of these samples were 6.01, 10.20, 6.26, 10.40, 8.86, and 9.72 μm, respectively (Table 1). Clearly, the average through-pore sizes of the PAN/PS was the smallest, and its through-pore size distribution was the narrowest. The much greater average through-pore sizes and wider distributions of PAN/PS W_1_, PAN/PS Co_1_, PAN/PS W_1_Co_1_, PAN/PS W_1_Co_2_, and PAN/PS W_1_Co_3_ are attributed to the increased AMT and Co(acac)_3_ which caused the increase of the diameter, as shown in Fig. 8.

**Table 1:** The detailed data of through-pore size distribution

Sample	Thorough-pore size distribution (μm)	Average thorough-pore size (μm)	Thorough-pore number (/cm^2^)
PAN/PS	5.56–6.62	6.01	7.73E+06
PAN/PS W_1_	9.57–17.10	10.20	1.39E+06
PAN/PS Co_2_	5.90–7.15	6.26	4.63E+06
PAN/PS W_1_Co_1_	9.62–11.40	10.40	1.28E+06
PAN/PS W_1_Co_2_	8.30–9.77	8.86	2.34E+06
PAN/PS W_1_Co_3_	9.18–10.70	9.72	1.74E+06

## Conclusions

In summary, the work described here demonstrated an efficient process to fabricate the AMT/Co(acac)_3_-loaded PAN/PS micro-nanofiber membranes with porous and large through-pore structure by one-step eletrospinning technique. The properties (morphologies, structures, element distribution, through-pore size, and through-pore size distribution) of these fibers were systematically investigated. Furthermore, the AMT/Co(acac)_3_-loaded PAN/PS significantly enhanced the through-pore distribution of relevant fiber membranes. The results showed the micro-nanofibers were successfully prepared and had potential applications in electrochemical reaction.

## Data Availability

All data generated or analyzed during this study are included within the article.

## Abbreviations

AMT: Ammonium metatungstate hydrate
Co(acac)_3_: Cobalt(III) acetylacetonate
Co., Ltd.: Limited company
Co_1_: Co^3+^ = 0.62 mmol
DMF: Energy disperse spectroscopy
EDS: Energy disperse spectroscopy
FeCo-CNFs: Iron and cobalt-incorporated carbon nanofibers
FESEM: Field emission scanning electron microscopy
FTIR: Flourier transformation infrared
PAN: Polyacrylonitrile
PS: Polystyrene
TPU: Poly (m-phenylene isophthalamide) (Nomex)/polyurethane
W_1_: W^6+^ = 0.62 mmol
W_1_Co_1_: W^6+^ = 0.62 mmol, Co^3+^ = 0.62 mmol
W_1_Co_2_: W^6+^ = 0.62 mmol, Co^3+^ = 1.24 mmol
W_1_Co_3_: W^6+^ = 0.62 mmol, Co^3+^ = 1.87 mmol
wt%: Weight fraction
W_*x*_Co_*y*_: W_1_ or Co_1_ or W_1_Co_1_ or W_1_Co_2_ or W_1_Co_3_
